# The complex effects of modern oncogenic environments on the fitness, evolution and conservation of wildlife species

**DOI:** 10.1111/eva.13763

**Published:** 2024-08-02

**Authors:** Antoine M. Dujon, Beata Ujvari, Sophie Tissot, Jordan Meliani, Océane Rieu, Nikita Stepanskyy, Rodrigo Hamede, Jácint Tokolyi, Aurora Nedelcu, Frédéric Thomas

**Affiliations:** ^1^ School of Life and Environmental Sciences Deakin University Waurn Ponds Victoria Australia; ^2^ CREEC/CANECEV (CREES), MIVEGEC, Unité Mixte de Recherches, IRD 224–CNRS 5290–Université de Montpellier Montpellier France; ^3^ School of Natural Sciences University of Tasmania Hobart Tasmania Australia; ^4^ Department of Evolutionary Zoology, MTA‐DE “Momentum” Ecology, Evolution and Developmental Biology Research Group University of Debrecen Debrecen Hungary; ^5^ Department of Biology University of new Brunswick Fredericton New Brunswick Canada

**Keywords:** ecotoxicology, environmental change, neoplasm, pollution, selective pressure

## Abstract

Growing evidence indicates that human activities are causing cancer rates to rise in both human and wildlife populations. This is due to the inability of ancestral anti‐cancer defences to cope with modern environmental risks. The evolutionary mismatch between modern oncogenic risks and evolved cancer defences has far‐reaching effects on various biological aspects at different timeframes, demanding a comprehensive study of the biology and evolutionary ecology of the affected species. Firstly, the increased activation of anti‐cancer defences leads to excessive energy expenditure, affecting other biological functions and potentially causing health issues like autoimmune diseases. Secondly, tumorigenesis itself can impact important fitness‐related parameters such as competitiveness, predator evasion, resistance to parasites, and dispersal capacity. Thirdly, rising cancer risks can influence the species' life‐history traits, often favoring early reproduction to offset fitness costs associated with cancer. However, this strategy has its limits, and it may not ensure the sustainability of the species if cancer risks continue to rise. Lastly, some species may evolve additional anti‐cancer defences, with uncertain consequences for their biology and future evolutionary path. In summary, we argue that the effects of increased exposure to cancer‐causing substances on wildlife are complex, ranging from immediate responses to long‐term evolutionary changes. Understanding these processes, especially in the context of conservation biology, is urgently needed.

## 1 INTRODUCTION

There is a growing acknowledgment that wildlife is becoming increasingly vulnerable to cancerous diseases compared to the past (Dujon, Ujvari, & Thomas, 2021; Giraudeau et al., 2018; McAloose & Newton, 2009; Pesavento et al., 2018; Sepp et al., 2019; Ujvari et al., 2018). Several examples of cancer in emblematic species include for instance the St. Lawrence beluga whales (*Delphinapterus leucas*) (Martineau et al., 2002), the marine green sea turtles (*Chelonia mydas*) (Dujon, Schofield, et al., 2021; Herbst, 1994), the California sea lions (*Zalophus californianus*) (Randhawa et al., 2015) as well as various fish species (Baines et al., 2021; Beamer et al., 1973). The explanation currently accepted for this abnormal increase in cancers is human activity, which has even recently led to humans being granted the status of “oncogenic species” for wildlife (Giraudeau et al., 2018). Such activities include both mutagenic pollution of the environment as well as changes in the diet and reductions in the genetic diversity (exacerbating problems of inbreeding) of wildlife species (Giraudeau et al., 2018; Ujvari et al., 2018; Sepp et al., 2019; Dujon, Schofield, et al., 2021). Exposure to mutagenic sources can alter a number of genetic and physiological processes in organisms, and it is important to understand the contribution of all processes to the phenotypic changes observed in each species. However, at least from a conservation point of view, it is important to go beyond factual observations and dissect precisely the diversity and the impact of all processes at work. Here, we argue that this is essential for assessing and predicting the impact of long‐term exposure to mutagenic contexts on the evolutionary trajectory of the species, with important consequences for wildlife conservation.

### 1.1 Increased cancer rates in wildlife species as a consequence of an evolutionary mismatch

Cancer arises from mutations within the body's cells, causing them to proliferate and spread, ultimately resulting in invasive tumours that can be costly or even lethal (Hanahan & Weinberg, 2011; Tomlinson et al., 1996). Cancer is a pathology that emerged with the advent of multicellularity (Aktipis et al., 2015; Albuquerque et al., 2018) and, in the vast majority of cases (with a few notable exceptions such as the devil facial tumour disease [DFTD] in Tasmanian devils *Sarcophilus harrisii*, Pye et al., 2016), it is not transmissible (see Dujon et al., 2020 for other examples). However, in contrast to other diseases, cancer cells themselves evolve during the lifetime, and within the environment, of the organism itself. In other words: (1) multicellular organisms have been under natural selection since their origins to develop and fine‐tune anticancer defences to reduce the fitness impact of this disease (Bissel & Hines, 2011; DeGregori, 2011); and (2) cancer cells have a limited window of evolutionary time, spanning a few years at most, and their evolution ceases (i.e., cancer goes extinct) with the death of their host (Arnal et al., 2015). Consequently, in an environment with stable oncogenic factors, it is expected that species will acquire through time appropriately anticancer mechanisms while cancer's evolutionary potential remains limited (i.e. co‐evolution is not possible), such that malignancies are rarely detrimental during an individual's reproductive phase. They are, however, expected to be more frequent and detrimental in post‐reproductive phases as the pressure of natural selection to maintain a high level of defence diminishes (Boddy et al., 2015; Frank, 2004).

It is currently accepted that the main reason why wildlife is presently developing more cancer than in the past is linked to human activities generating increasing oncogenic risks, for which the previously evolved anti‐cancer defences are now inadequate (e.g. Baines et al., 2021; Dujon, Schofield, et al., 2021; Giraudeau et al., 2018; Sepp et al., 2019). Such discrepancies are generally referred to as “evolutionary mismatches” (Lloyd et al., 2011). The unsurprising corollary is that species living in environments more conducive to cancer development than those in which they primarily evolved will display higher cancer rates before or during their reproductive periods (Dujon, Ujvari, & Thomas, 2021; Michael & Noble, 2017) (Box 1). For instance, species such as humans or dogs are particularly prone to cancers because there are numerous and recent differences between their ancestral and current environmental conditions and lifestyles (diet, morphology, reproductive regimen, etc.) while their anti‐cancer defences have remained unchanged (Aktipis & Nesse, 2013; Leroi et al., 2003; Sarver et al., 2022).

BOX 1 **: Different scenarios detailing the effect of oncogenic pressure on the life‐span of an individual and the observed prevalence of cancer in wildlife populations**

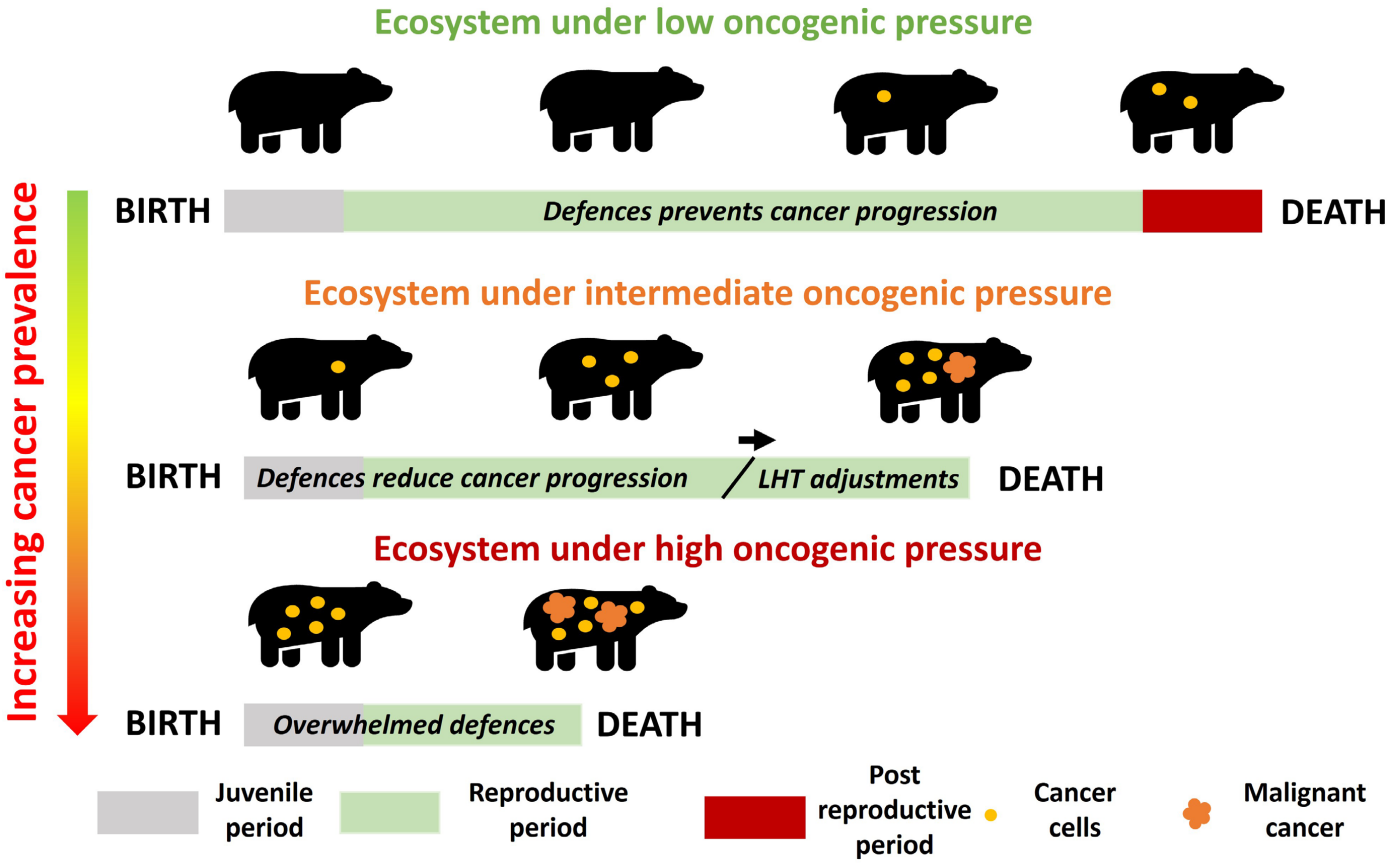

Different scenarios should be considered when wildlife is exposed to new cancer risk factors. In an ecosystem with stable oncogenic pressures, anti‐cancer defences are optimized over multiple generations by natural selection to prevent the occurrence of tumours during the reproductive period, adjusting the trade‐offs between reproduction and anti‐cancer defences (Dujon et al., 2023; Dujon, Boutry, Tissot, Lemaître, et al., 2022; Jacqueline et al., 2017; Thomas et al., 2019). As a consequence, in undisturbed ecosystems, the prevalence of tumours is predicted to be relatively low and affect mostly individuals in post‐reproductive years (see the example of mammals, Vincze et al., 2022). In ecosystems in which oncogenic pressures begin to increase, the prevalence of tumours in wildlife, but not necessarily cancer‐induced mortality, is expected to increase (e.g. as observed in the pandemic of sea turtles fibropappillomatosis, Dujon, Schofield, et al., 2021).The life span of animals may be reduced by the increased energetic cost of consistent activation of anti‐cancer defences, but over most of the reproductive period they will still prevent the large majority of cancers to progress to the lethal stage. The various studies investigating habitat degradation in terrestrial and aquatic ecosystems suggest a large proportion of Earth's ecosystem are in that scenario (Gibbs & Salmon, 2015; Halpern et al., 2008; Tang et al., 2021). In highly disturbed ecosystems, with high and novel oncogenic pressures, anti‐cancer defences are insufficient to prevent cancer emergence and progression (see the Beluga whale population in the St. Lawrence, Martineau et al., 2002). Tumours are predicted to be widespread, significantly contributing to animal mortality if the animal is allowed to live long enough without dying from other extreme environmental disturbances. Such situation are currently occurring on local scales, in highly polluted habitats (Baines et al., 2021; Møller et al., 2013). In the long term, for species that will not go extinct, it is expected that other adjustments will be made in anti‐cancer responses, from the adjustment of life history traits (LHT in the above figure) or tolerance, to the selection of anti‐cancer mechanisms more in line with the increased frequency of cancer risk.

Among the most prominent examples highlighting the rapid and sudden increase in the risk of mutagenic and therefore carcinogenic effects of modern human activities is the massive release of radioactive isotopes in the air, soil and water in the regions of Chernobyl in Ukraine and Fukushima in Japan, following their nuclear accidents in 1986 and 2011, respectively (Steinhauser et al., 2014). Other examples worth mentioning are the sites of nuclear weapon tests such as the Bikini Atoll. While immediate exposure to radiation following the release of radioactivity in the environment negatively impacted the health of local species, decades later, species who were able to resist the radiations in the environment thrive, suggesting they may have evolved stronger anti‐cancer defences (Dillon et al., 2023; Richards et al., 2008; Webster et al., 2016). This is however not the case for all species and underlines the need for further research on the subject (see Beresford et al., 2020; Beresford & Copplestone, 2011; Cunningham et al., 2021). These cases are particularly noteworthy because of their extreme impact, but also because they represent only the visible part of a wider problem. Human activities are responsible for many less spectacular mutagenic effects, but they should not be trivialised, because the consequences of chronic low‐dose exposure are important to consider.

**FIGURE 1:**
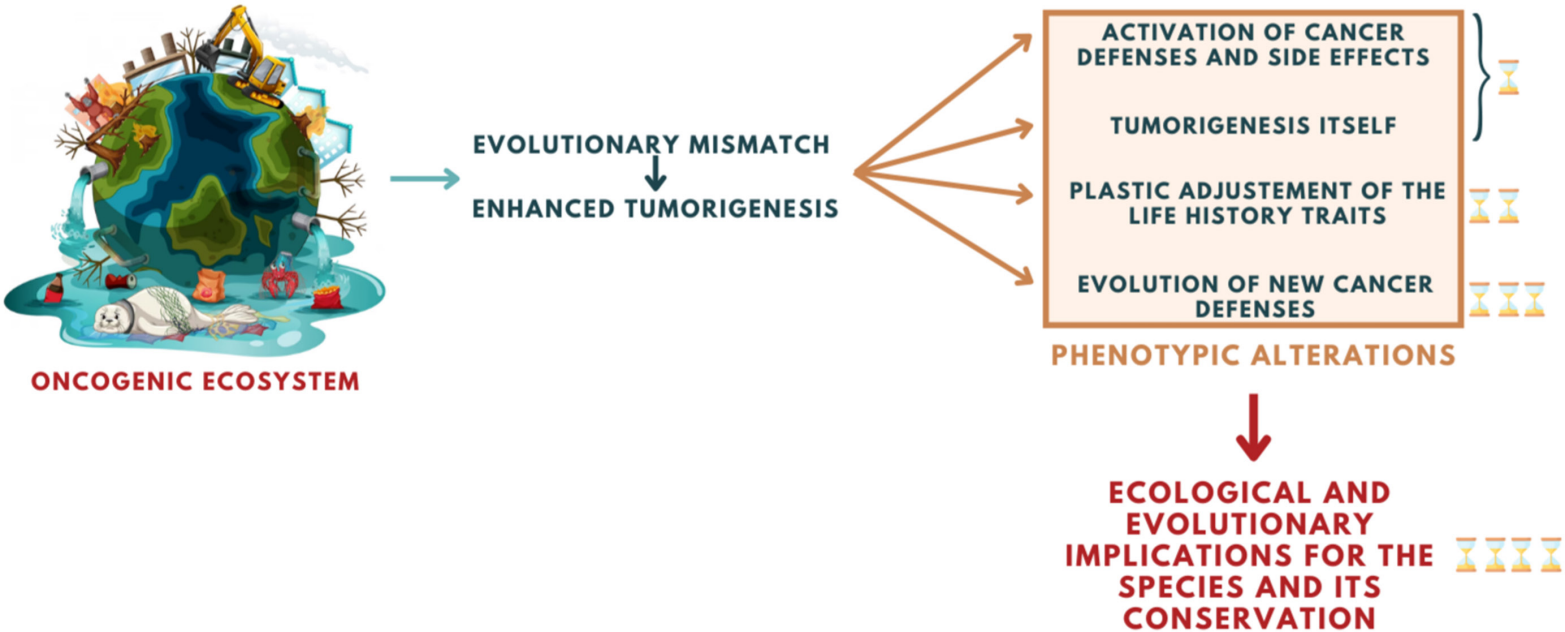
Summary of the oncogenic consequences of human activities on wildlife. Wild species are increasingly faced with evolutionary mismatches because their ancestral anti‐cancer defences have not been tuned to be powerful enough in relation to the currently enhanced cancer risks. This has several consequences: (i) In the short term, the anti‐cancer defences are over‐activated, an adaptive response that can now be detrimental because of the costs of each activation, in terms of both energy and tissue homeostasis. (ii) When these defences fail, early tumorigenesis itself can have indirect effects that have repercussions on the phenotype and fitness of the hosts. (iii) In the medium term, species may adapt by adjusting their life history traits, for instance by maximising immediate reproductive episodes. (iv) In the long term, some species are able to select more powerful anti‐cancer defences. All these processes, interacting with other selective constraints in ecosystems, help to shape the evolution of species in anthropised ecosystems, which in turn determines their sustainability.

All species are inhabiting an ever‐changing landscape in which multiple cancer risk factors (including those associated with increased oncogenic human activities) interact, causing wild species to adjust their level of defences to the new risks (Dujon, Ujvari, & Thomas, 2021). Here, we argue that from both evolutionary and conservation biology perspectives, we must understand (i) how quickly these adjustments can happen in the face of constant and rapid degradation of ecosystems, and (ii) what are the consequences of these adjustments on the life history and evolution of wildlife species as well as on the ecosystem itself (Dujon, Aktipis, et al., 2021). To do this, we need to appreciate the short‐, medium‐ and long‐term phenomena that occur in organisms and species that suddenly find themselves in an oncogenic context that is higher than the one in which their anti‐cancer defences evolved. These include: (i) the cost of activating anticancer defences (and the side effects), (ii) the cost of tumorigenesis itself, (iii) changes in life history traits and (iv) selection for improved anti‐cancer mechanisms. The new possible evolutionary trajectories that wildlife species can follow will reflect the combination of these effects with the other selective constraints exerted by biotic and abiotic conditions in ecosystems (Figure 1).

### 1.2 The cost of activating anticancer defences

The anti‐cancer defences of an organism encompass multiple layers intended to stop the initiation and progression of malignancies at different stages (Box 2). Regardless of the nature of the defences activated, none of them are ultimately without costs; they use energy which cannot be allocated in the other body functions because of trade‐offs (Boutry et al., 2020; Jacqueline et al., 2017; Klaasen et al., 2024). In addition, energy is not the sole cost: apoptosis and/or cellular senescence, which are powerful protective mechanisms against tumorigenesis (DeGregori, 2011; Wang et al., 2022), are not very costly in terms of energy, but they remove cells that could be involved in normal homeostasis, resulting in indirect costs (Aktipis, 2020; Tower, 2015). In an environment that is rapidly and abnormally becoming more mutagenic, the frequency of oncogenic processes may increase considerably, leading to the systematic activation of the defences aimed to eliminate them. However, the increased cost of that systematic activation is predicted to shift evolutionary trade‐offs and to have a significant negative impact on traits like survival and/or reproduction (Dujon, Boutry, Tissot, Lemaître, et al., 2022). To appreciate the potential importance of this phenomenon, it is striking to note, for example, that even after a single immune challenge, the consequences of activating defences can last for days or even weeks (see the example of the blackbird, Box 2). Thus, at medium level of exposure to risk factors, we can predict that species exhibiting this type of response may not necessarily have visible cancers, but rather be in a state of serious exhaustion because of their natural tendency to use costly defences to eliminate an enemy that has become abnormally frequent. This phenomenon can be considered as an evolutionary mismatch, since it is now maladaptive, with anti‐cancer defences that evolved in ecosystems with low oncogenic backgrounds now constantly activated, consuming resources that cannot be invested in other body functions such as reproduction (see Robertson & Blumstein, 2019; Schlaepfer et al., 2002). It is surprising that this process has not yet been seriously considered in the context of cancer defences, even though it has been clearly documented in other conceptually similar contexts. For instance, such responses are well‐known with costly detoxification adaptations in polluted areas (e.g. carabid beetles, woodlice or ragworms exposed to heavy metals, Jones & Hopkin, 1998; Pook et al., 2009; Stone et al., 2001), as well as in the context of host–parasite interactions. For the latter, energetically expensive defence strategies that are effective in the case of sporadic infections may become unsustainable in the case of very frequent infections, because the cost of the defences mortgages the energy allocated to other functions (Boots & Haraguchi, 1999; Stjernman et al., 2008; Walsman et al., 2023). In addition, the overactivation of immune defences can lead to other pathologies such as autoimmune diseases, which can be highly detrimental to fitness (De Lisle & Bolnick, 2021; Vrtílek & Bolnick, 2021). Potentially similar harmful consequences are expected in the event of excessive activation of immune defences in the face of a recurrence of cancer cells (Aktipis, 2020; Giat et al., 2017). This must be explored in the context of wildlife. For instance, fibroblasts extracted from bank voles residing in the vicinity of the Chernobyl nuclear power plant accident site exhibit heightened antioxidant levels, reduced susceptibility to apoptosis, and enhanced resilience against oxidative and DNA‐related stressors (Mustonen et al., 2018). The direct and indirect costs of these cellular attributes that probably contribute to the bank voles' ability to adapt to the radioactive environment are not known at the moment. Costs may also depend on the initial physical condition of the exposed individuals, and be amplified in individuals of poor quality (see, for example, Boratyński et al., 2021). Admittedly, this situation may evolve over time, so that other, more appropriate strategies take over (e.g. tolerance or adjustment of life‐history traits in response to cancer pressure). However, even if these adaptations do exist in relation to cancerous processes (see below), it is not yet known how easily they can be implemented in a species that is not used to this type of response.

BOX 2 **: Anticancer defences are a multilayer system that can be costly**

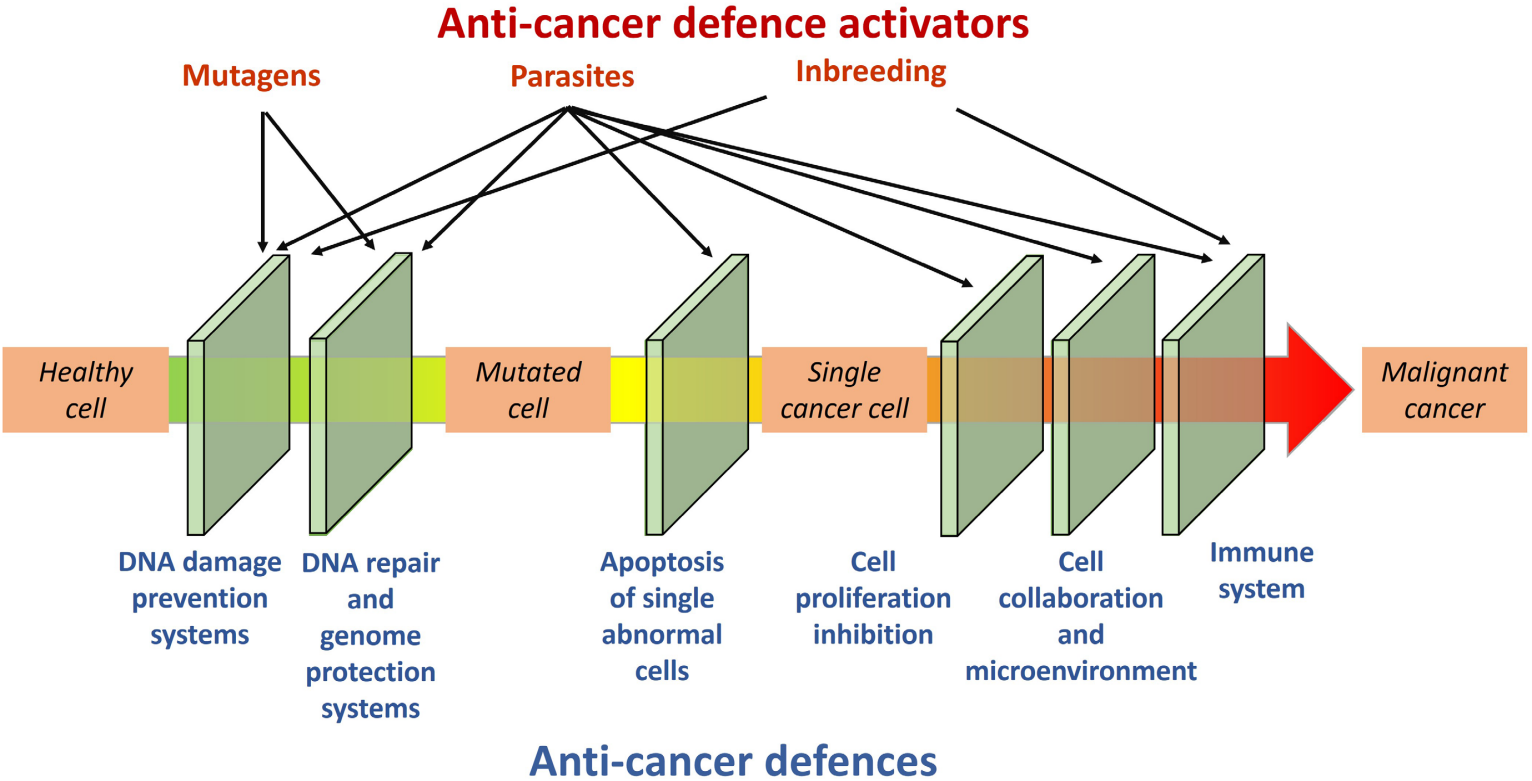

All lineages evolved powerful ways to prevent or deal with the consequences of DNA damage and deleterious mutations. In the context of cancer, these mechanisms are known as cancer or tumour suppression mechanisms. Still, DNA can be damaged by reactive oxygen species (ROS) (Benzie, 2000), radiations or parasites, which induce single or double strand breaks for the former, or the formation of cyclobutane pyrimidine dimers for the later (Cadet et al., 2005); when not repaired properly, the damage can result in mutations with oncogenic potential. However, ROS can also activate various anti‐cancer defences. For instance, antioxidant systems quench, scavenge, divert or bind reactive oxygen species preventing them from damaging the DNA (Benzie, 2000). Nevertheless, the over activation of these systems can be energetically costly, and potentially interfere with other cellular processes. In the majority of cases, the accumulation of mutations or too much damage in a cell's DNA leads to cell cycle arrest or the activation of the apoptosis pathway (Niida & Nakanishi, 2006). Yet, these processes reduce the number of functional cells, and if they affect stem cells, have the potential to reduce the lifespan of the individual (i.e. a double‐edge sword, Shen & Tower, 2009). If a cancer cell is able to avoid apoptosis, for example because the p53 gene is mutated (Goh et al., 2011), it has to enable infinite replicative potential, break the physicochemical constrains of its microenvironment but also ecological constrains of the surrounding healthy cells (e.g. as observed in the epithelial‐to‐mesenchymal transition, Roche, 2018). Again, maintaining a healthy tissue with a competitive advantage relative to oncogenic cells requires diverting energy into somatic maintenance, and potentially diverting it from reproduction. Another key step to become fully malignant and gain the ability to invade other organs is the acquisition of the ability to evade destruction by the immune system (Vinay et al., 2015). It is currently unknown to what extent the activation of the immune system by cancer cells increases energetic expenditure. As a point of reference, immune challenges showed that the activation of the immune system by a pathogen's antigen increases the metabolic requirement by 5%–15% (Hasselquist & Nilsson, 2012) with an effect on the behaviour of the animal that can last for weeks (e.g. three weeks in free‐living Eurasian blackbirds, *Turdus merula*, Lennon et al., 2023). The efficacy of each anti‐cancer defence can be simultaneously or independently degraded by pressures applied on the host species usually forming a complex cancer risk landscape (Dujon, Ujvari, & Thomas, 2021). For example, a large proportion of cancers are initiated by parasitic infections because they damage the DNA directly (e.g. retroviruses Desfarges & Ciuffi, 2012) through oxidative stress (Kawanishi et al., 2016), and more importantly because they directly interfere with the tumour suppressors genes that control apoptosis, cell cycle arrest, telomerase expression, and cell adhesion (Ewald et al., 2015; Ewald & Swain Ewald, 2019). The efficacy of the immune system in eliminating both parasites and cancer cells themselves is dependent on the genetic diversity of the host. Species that went through a genetic bottleneck due to human activities are likely to be under increased risk of developing malignant cancer (as observed in the Tasmanian devil with DFTD and the Californian sea lion, *Zalophus californianus*, with carcinoma, Browning et al., 2015; Cheng et al., 2012). Animal in degraded habitats, for example urban habitats, exhibit higher oxidative stress and increased inflammation levels, a condition promoting the proliferation of cancer cells (Isaksson, 2015; Nath et al., 2010).

Overall, the anti‐cancer defences of an organism are multilayered, protecting the animal from spatial scales smaller than a cell up to a whole organ. Each defence layer evolved over multiple generations through selection optimising the trade‐off between energy expenditure invested in the various anti‐cancer defences and other body functions (e.g. daphnias have to balance resistance to UV radiations, swimming performance to avoid predation, and reproductive effort, Sha et al., 2020; Sha & Hansson, 2022). This process is based on selective pressures applied by the environmental conditions a generation inhabits. Ultimately, species might evolve better or new cancer suppression mechanisms, including, for instance, the development of skin pigments, fur, a black peritoneum or a nocturnal life style which are efficient ways to protect against damage caused by the UV emitted by the sun (Burtt, 1981; Trosko, 2001).

To sum up, the first thing that needs to be considered in order to understand the biology of species that are newly confronted with abnormal mutagenic contexts, is the cost of activating anti‐cancer defences. To explore this field of research, it could be appropriate to use first models in which we can simulate the appearance of oncogenic mutations (e.g. see for instance Fortunato et al., 2021; Klaasen et al., 2024) or, even better, cancer, for example by adapting the technologies developed by medical research into anti‐cancer vaccines (Saxena et al., 2021; Schumacher & Schreiber, 2015) or through activating certain oncogenic pathways using specific mutagens (Dujon, Boutry, Tissot, Meliani, et al., 2022). By designing a challenge that exposes animals to proxies of cancer inducers, with the aim of triggering their anti‐cancer defences without inducing malignant tumor growth, we can effectively distinguish the effects of activating anti‐cancer defences from the underlying pathology (see next point).

### 1.3 The consequences of tumorigenesis itself

It is well known that cancer in its late stages is highly damaging to health and therefore has negative effects on performance in relation to fitness‐related variables. However, less is known about the direct effects of early stages of tumorigenesis (Thomas et al., 2017, 2018). Cancer cells, because of their proliferation and their particular biology, have the capacity to impose fitness costs to their hosts in all stages of cancer progression (Ujvari et al., 2016). But we are still far from being able to assess, depending on the species and the type of cancer (i.e. the organ concerned), the effect that tumourigenesis (throughout its continuum from precancerous lesions to metastatic cancers) has on fitness‐related parameters (e.g. competitive aptitude, ability to escape predators or to hunt, resistance to parasites, dispersal capacity, etc… Boutry, Mistral, et al., 2022; Dawson et al., 2018; Duneau & Buchon, 2022; Hamilton et al., 2020; Makin et al., 2021; Vittecoq et al., 2013). For these questions, it seems essential to use biological models in which it is possible to experimentally induce tumorigenesis, and to follow the links between the changing characteristics of the tumour and the concomitant changes in the host phenotype through time (i.e. depending on species, days, weeks, months and years that follow, Dujon, Boutry, Tissot, Meliani, et al., 2022). If the models used are the same as those envisaged in the previous section, it will even be possible to separate the effect of tumorigenesis alone on the phenotype, i.e. without the cumulative effect of the activation of anti‐cancer defences. Alternatively, another approach to tackling this question involves grafting cancerous cells into an animal that has not previously been subjected to an oncogenic environment. In all cases, the progression of the disease will have a major impact on the physical condition and hence the fitness of the individuals concerned, and all the more so if we consider wild species which must also be confronted with problems of predation, competition or sexual selection. If the propensity to develop cancer in a mutagenic environment is also influenced by the genetic background of individuals, the evolutionary consequences within populations will be changes in the frequency of certain genetic variants.

### 1.4 Modification of life history traits

Faced with an evolutionary constraint for which there is no direct solution of elimination or escape, natural selection favours modifications to life history traits (when possible), aimed at reducing the impact of the constraint on the selective value (e.g. Brannelly et al., 2021; Dasgupta et al., 2022). This type of response, well known in the context of host–parasite interactions (Agnew et al., 2000; Michalakis & Hochberg, 1994), has also been documented with cancerous processes: in Tasmanian devils (Jones et al., 2008), *Drosophila* (Arnal et al., 2017) and hydra (Boutry, Tissot, et al., 2022), where a maximisation of the reproductive effort is observed before the animals are strongly impact by the disease. For Tasmanian devils, precocial breeding is a response of increased availability of resources and faster growth rates as a result of DFTD‐induced population decline, whereas in *Drosophila* and hydra it is an individual plastic response (i.e. displayed only by tumour‐bearing individuals). This response is also observed in certain species exposed to well‐identified cancer risk factors without the development of tumours, such as daphnia or marine copepods, which rapidly increase their reproductive effort when exposed to UV radiation (Heine et al., 2019; Sha et al., 2020). Similarly, parasites transmitted through the environment could illustrate these phenomena, as they spend time in non‐living surroundings where they encounter various stressors, including mutagenic ones (Rogalski & Duffy, 2020). Strains of parasites obtained from lakes with better sunlight penetration (clearer water), displayed the greatest resilience to the detrimental consequences of sunlight exposure. This suggests that they have adapted to thrive in sunnier conditions. This adaptation, however, carried both advantages and drawbacks for the parasites: strains from these clearer lakes generated comparatively fewer transmission stages (spores), but they exhibited higher infectivity. Following experimental exposure to sunlight, the parasite strains most tolerant to sunlight decreased host fecundity to the same extent as spores that had never experienced sunlight exposure.

Clearly, further research is needed to determine how easily and/or quickly population‐level and/or individual responses to cancer risks via life history traits adjustments can be favoured (through the effects of epigenetics, phenotypic plasticity, and genetics each occurring at different temporal scales). One could hypothesize that this might not demand an extensively long period, as cancers affecting the reproductive phase have traditionally been rare in numerous species (owing to effective defences, as discussed earlier). However, the immediate impacts it could induce in present circumstances might resemble those of other prevalent animal pathologies (such as infectious diseases), for which the activation of plastic responses over the lifespan has already been favoured by selection. Generally speaking, when a tumour causes all or part of the effects of another more common pathology, it may be enough for the responses selected for these pathologies to be triggered as well, even if the underlying health problem is different. Possibly in accordance with this statement, Stepanskyy et al. (*in preparation*) have shown that the cnidarian *Hydra oligactis* from the wild, known to often develop spontaneous tumours when placed in the lab, enhance their budding rate before tumours develop. Such a response, previously detected in hydra bearing vertically transmissible tumours for years (see Boutry, Tissot, et al., 2022), was interpreted as the possible result of coevolution between the host and the tumours through time. In the light of these new results obtained with the wild hydra, it seems that the spontaneous tumours could instead be mimicking a transmissible disease that has an impact on reproductive potential, for which the hydra has already selected an adaptive plastic response in order to mitigate the cost on fitness.

An important direction in this research area is also to understand why some wild species do not seem to be able to adjust their life‐history traits when living in environments that have become highly mutagenic. This is the case, for example, of the crustacean *Asellus aquaticus* that live in the Chernobyl region: according to Robertson and Blumstein (2019), the current dose rates at Chernobyl are not causing discernible effects on the reproductive output of *A. aquaticus* (see Fuller et al., 2018 and also Beresford et al., 2022; Burraco et al., 2021). It remains uncertain whether this species possessed robust preexisting anti‐cancer defences, enabling it to mitigate the impacts of harmful radiation, thus showing no effect on life history traits. Alternatively, it's possible the species evolved resistance to radiation following exposure, with adjustment of life history traits, the consequences of which are only observed now (i.e. the evolutionary process was not recorded). It also seems important to extend the study of life history traits to behavioural aspects, since they can help reduce exposure to mutagenic substances, as has been shown. For example in Chernobyl Great tit *Parus major* and pied flycatcher *Ficedula hypoleuca* are capable of selecting the least contaminated areas for their reproduction either in response to the direct exposure or because food sources got displaced by radiations (Møller & Mousseau, 2007).

### 1.5 Selection for improved anti‐cancer defences

If exposure to mutagenic environmental factors persists for long periods, natural selection could confer a selective advantage on individuals with more powerful anti‐cancer defences. An intriguing line of research aimed at predicting the outcome of such selection involves studying the cancer‐suppressing mechanisms that may have evolved in species and/or populations that have lived in naturally mutagenic environments for a long time (Vittecoq et al., 2018). For example, *Drosophila melanogaster* living at high altitudes have specific adaptations to counter the ultraviolet radiation that damages DNA. These adaptations include genetic variations within DNA repair genes (Svetec et al., 2016). Similarly, the marine mussel *Bathymodiolus azoricus* inhabiting volcanic vents, an environment with a genotoxic cocktail of high pressures, temperatures, radionucleides, hydrogen sulphides and heavy metals recover quickly from DNA damages (Pruski & Dixon, 2003). Furthermore, we may discover anti‐cancer adaptations similar to those described in species whose theoretical susceptibility to oncogenic processes derives from other factors, such as large size and/or longevity (Vincze et al., 2022). For example, the cells of African and Asian elephants, particularly the species *Loxodonta africana* and *Elephas maximus*, show increased reactivity to DNA damage resulting in higher rates of apoptosis due to the presence of 20 duplications of the tumour suppressor gene TP53 in their genome (Abegglen et al., 2015; Sulak et al., 2016). In the context of the long‐lived whale *Balaena mysticetus*, (Keane et al., 2015) uncovered a multitude of mechanisms that suppress cancer. These mechanisms include positive selection of various ageing‐ and cancer‐related genes (e.g. ERCC1, a pivotal gene in DNA repair pathways), as well as gene duplications associated with DNA repair (e.g. PCNA) and cell growth regulation (e.g. LAMTOR1 also explored by Tollis et al., 2019, concerning *Megaptera novaeangliae*). Interestingly, such mechanisms might not necessarily protect these large and long‐lived animals from environmentally‐induced cancers, as Beluga whales inhabiting polluted waters have an increased rate of cancer (Martineau et al., 2002) suggesting their protective effects are specifically tuned to mitigate the increase in cancer risk predicted by an increase body size and longevity and may be inadequate to face novel oncogenic threats. More research is needed to evaluate the real cost and benefit of additional cancer defences, especially when cancer result from exposure to mutagens. For instance, Moding et al. (2016) found in mice that an extra copy of *p53* blocks the development of spontaneous Kras‐driven lymphomas and lung cancers, but not radiation‐induced lymphomas.

Another aspect to consider in understanding the evolution of anti‐cancer defences is their *net* effect on the selective value of an individual, not just their ability to reduce and/or eliminate cancerous progression. As mentioned above, anti‐cancer adaptations are not without costs, and in “Darwinian currency” it is the final result on selective value that counts. In addition to the costs already mentioned, it is therefore important to also consider the selective landscape as a whole, taking into account all the players in the ecosystem (including humans, see for instance Deryabina et al., 2015) as well as the ecological status of the species in question. For example, the presence of predators could prevent the selection of additional anti‐cancer defences in a prey species if they are accompanied by costs that considerably increase the probability of predation. An indirect approach to explore this question involves studying the sometimes remarkable anti‐cancer adaptations that domesticated species have occasionally developed (Thomas et al., 2020). The selection of those adaptations can be attributed to the significant mismatch disparities arising from domestication conditions, breeders' incentives to maximize animal survival, and the absence of interactions such as competition and predation, which could have led to overly costly solutions in natural ecosystems.

### 1.6 Concluding remarks

In the light of the information discussed here, it appears that several distinct processes can generate phenotypic changes in organisms that find themselves in an evolutionary mismatch with regard to vulnerability to cancer (Figure 1). These effects, which shape the evolutionary ecology of species, are not mutually exclusive. For example, some Tasmanian devils are now resistant, i.e. able to eliminate the tumour (see Epstein et al., 2016, although the cost of this resistance is not yet known), others seem to be tolerant (Ruiz‐Aravena et al., 2018), while others, as mentioned above, adjust their life‐history traits (Jones et al., 2008). An important research question to explore is whether the adjustment of life history traits is easier to select for than the development of improved resistance to cancer (Thomas et al., 2019). If the oncogenic pressure becomes increasingly strong in ecosystems, it is also possible to think that adjusting life history traits will not be enough to preserve the species in the long term, and that sooner or later the species will have to develop superior anti‐cancer defences or risk extinction. For example, in the case of Tasmanian devil populations heavily infected by the transmissible cancer DFTD, individuals are now able to reproduce in their first year, but it is not possible to reduce this delay any further because of seasonal constraints. Moreover, over 60% of females can only engage in a single reproductive event before succumbing to DFTD (in contrast to nearly 90% of females before the epidemic) with little possibility for life history trait adjustment after this first reproduction event (Jones et al., 2008). It is therefore possible that the selection for resistance is only now favoured by selection, and that the adjustment of life‐history traits has been a transitional solution, possibly even slowing down the selection for resistance sensu stricto beforehand.

As well as already being a major area of research in conservation biology, the ideas proposed in this paper could also have a major impact within the community of ecologists and evolutionary biologists. Indeed, a crucial notion for these disciplines is inter‐individual variability and its origin. Until now, inter‐individual differences have often been attributed to the genes of individuals, or to variables such as parasitism. It is possible that part of the explanation not yet considered is linked to the presence of sub‐clinical stages of tumours (Pineda‐Krch & Lehtilä, 2004). For example, everyone will probably agree that a metastatic stage cancer will explain almost 100% of the variance in performance compared to individuals without a tumour. On the other hand, no‐one has really looked into this question for the early stages of tumourigenesis, when the effects of tumourigenesis are unlikely to suddenly manifest themselves until the end of the continuum. The same reasoning can be applied to the activation of anti‐cancer defences. For example, if some individuals have a greater basic vulnerability than others to developing cancer and the environment becomes oncogenic, these same individuals will have different levels of activation of their anti‐cancer defences. The repercussions of the costs generated via the associated trade‐offs will generate inter‐individual variability, with possibly transgenerational effects. The evolutionary consequences of the inter‐individual variability generated by these processes are not yet known and will very likely be different depending on their ecological status, whether for instance it is a prey or predator species, as weakened prey in ecosystems are often captured more easily. It is thus worth noting as well that while our focus here was on the direct costs of activating anti‐cancer defences, species with activated defences will also interact with non‐oncogenic risk factors and other environmental traits within the complex network of interactions that form ecosystems. These interactions could either exacerbate or alleviate the costs of anti‐cancer defences, but they could also obscure them, potentially explaining why they have been overlooked. Multiple cascading effects are possibly expected, and the effects of the activation of anti‐cancer defences may in combination with other stressors represent a tipping point for species. Clearly these aspects remain poorly understood to date (Dujon, Brown, et al., 2021; Roche et al., 2017).

In the light of this article, it seems evident that we cannot simply assume that species inhabiting ecosystems now more prone to mutation will merely experience increased rates of cancer and subsequent population declines due to associated mortality. Clearly, the biology of host‐tumour interactions in our changing world is more complex, and it is highly topical for the sciences of evolutionary ecology and conservation to explore these research themes. Such research will additionally contribute to a more comprehensive evaluation of the role cancerous processes have played in the evolution of multicellular organisms throughout their entire existence.

## ACKNOWLEDGEMENTS

None.

## FUNDING INFORMATION

This work was supported by the Hoffmann Family, a CNRS International Associated Laboratory Grant, the ANR EVOSEXCAN project (ANR‐23‐CE13‐0007). This work was also carried out within the framework of the Camargue Health‐Environment “Zone Atelier” (ZACAM) of the “Long‐Term Socio‐Ecological Research network” (LTSER‐RZA), funded by the Ecology & Environment of the French National Center for Scientific Research (EE‐CNRS).

## CONFLICT OF INTEREST STATEMENT

Frédéric Thomas is an Editorial Board member of Evolutionary Applications and a co‐author of this article. To minimize bias, Frédéric Thomas was excluded from all editorial decision‐making related to the acceptance of this article for publication.

## Data Availability

Data sharing not applicable to this article as no datasets were generated or analysed during the current study.
